# Evaluation of Hirst-type spore traps in outdoor *Aspergillaceae* monitoring during large demolition work in hospital

**DOI:** 10.1371/journal.pone.0191135

**Published:** 2018-01-18

**Authors:** Sophie Tiphaine Loeffert, Philippe Vanhems, Estelle Tissot, Cédric Dananché, Pierre Cassier, Thomas Bénet, Michel Perraud, Michel Thibaudon, Marie-Paule Gustin

**Affiliations:** 1 Laboratoire des Pathogènes Emergents-Fondation Mérieux, Centre International de Recherche en Infectiologie (CIRI), Inserm U1111, CNRS UMR5308, ENS de Lyon, France, Université de Lyon 1, France; 2 Unité d'hygiène, épidémiologie et prévention, Groupement hospitalier Edouard Herriot, Lyon, France; 3 Analyzair, Lyon, France; 4 Laboratoire de Biologie Sécurité Environnement, Groupement hospitalier Edouard Herriot, Lyon, France; 5 Réseau National de Surveillance Aérobiologique, Brussieu, France; 6 Département de santé publique, Institut des Sciences Pharmaceutiques et Biologiques (ISPB)-Faculté de Pharmacie, Université de Lyon 1, France; Ecole des Mines d'Ales, FRANCE

## Abstract

Demolition can generate fungal spore suspensions in association with various adverse health effects, such as high risk of invasive aspergillosis in immunocompromised patients. One block of Edouard Herriot Hospital was entirely demolished. The aim of the present study was to evaluate Hirst-type spore traps utility in monitoring outdoor *Aspergillaceae* (*Aspergillus* spp. + *Penicillium* spp.) spores in part of Edouard Herriot Hospital (Lyon, France) undergoing major demolition. Three periods were scheduled in 2015: (A) Gutting of building and asbestos removal, (B) Demolition of floors, (C) Excavation and earthwork. Outdoor *Aspergillaceae* fungal load was monitored by cultivable (Air Ideal®, bioMérieux) and non-cultivable methods (Lanzoni VPPS-2000, Analyzair®, Bologna, Italy). Differences of *Aspergillaceae* recorded with Hirst-type spore traps were observed between Gerland and Edouard Herriot Hospital. Differences between *Aspergillaceae* were recorded between day time and night time at Gerland and Edouard Herriot Hospital. Daily paired differences between *Aspergillaceae* recorded with non-cultivable methodology at Edouard Herriot Hospital and in an area without demolition work were significant in Period A vs Period B (p = 10–4) and Period A vs Period C (p = 10–4). Weak correlation of daily *Aspergillaceae* recorded by both methods at Edouard Herriot Hospital was significant only for Period C (r = 0.26, p = 0.048, n = 58). Meteorological parameters and type of demolition works were found to heavily influenced *Aspergillaceae* dispersion. Non-cultivable methodology is a promising tool for outdoor *Aspergillaceae* scrutiny during major demolition work in hospital, helping infection control staff to rapidly implement control measures.

## Introduction

Healthcare establishments are frequently confronted by infrastructure work. Construction work, particularly demolition, generates 10^5^-fold elevated outdoor levels of thermo-tolerant fungi compared to non-demolition values and, consequently, augments the risk of invasive aspergillosis (IA).in immunocompromised patients, a severe infection with 50–90% lethality caused mostly by *A*. *fumigatus* (>80%)[1–3]. Specific guidelines have been published to increase indoor hospital air control measures during construction [4]. A pilot study, conducted at EHH in 2013, evaluated the use of Hirst-type spore traps (HTSTs) in environmental fungal load monitoring of indoor and outdoor hospital units [5]. This work showed that indoor HTSTs were not useful but confirmed previous results, suggesting that indoor fungal contamination is highly influenced by outdoor, airborne spores [6]. Large-scale surveillance systems are needed to detect outdoor fungal spores and alert hospitals to quickly implement control measures. Surveys with spore traps are required to develop such warning systems [7].

Some studies have evaluated the reliability of different sampling methods (cultivable and non-cultivable) in measuring fungal spore concentrations [8–14]. Each sampling method seems to have advantages and drawbacks. Non-cultivable methods allow the sampling of numerous spores, useful to carry out surveys. However, fungal spore’s identification only possible at the genus level [15]. Cultivable methods allow spore identification at the species level but is time-consuming and depends on the substrate plated and culture conditions applied [10]. Other factors, such as environment and meteorological parameters, may also affect the survivability of fungal spores [11,14]. To the best of the authors’ knowledge, no standardized method of *Aspergillus-Penicillium* (*Aspergillaceae*) assessment has been developed by comparing results with paired samples [9,11,16].

The main objective of the present study was to evaluate the utility of HTSTs for outdoor *Aspergillaceae* monitoring during large demolition work in hospital. The secondary objectives were to correlate the results of HTSTs with an agar impact sampler and to evaluate the impact of meteorological parameters on *Aspergillaceae* aero-contamination measured by both methods.

## Materials and methods

### Study sites

**Fig 1:**
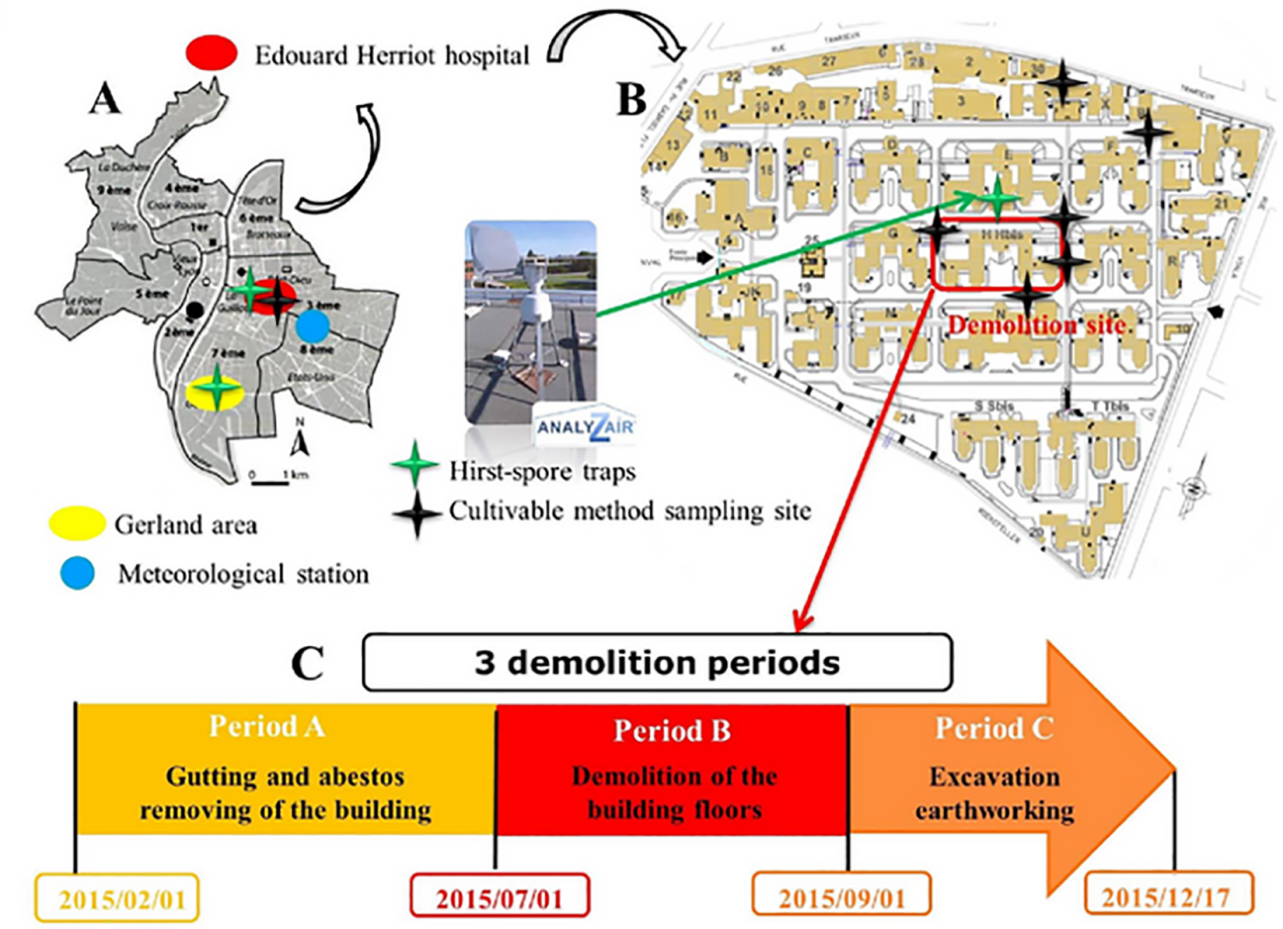
Study site. (A) Sampler locations in Lyon, France, (B) Edouard Herriot Hospital, and (C) demolition work schedule.

This study was conducted at University of Lyon-affiliated Edouard Herriot Hospital (EHH, 850 beds) and at Gerland area located a few km south of the hospital and subject to similar weather conditions (Fig 1A). An entire block, in the center of EHH, was entirely demolished. The time period was separated into 3 consecutive periods between February and December 2015 (Fig 1B and 1C, video of the demolition work: https://www.youtube.com/watch?v=Oa7xRufAnhQ). No major demolition work was undertaken in proximity to the Gerland area which served as control site.

### Aspergillaceae monitoring

Airborne fungal sampling was undertaken simultaneously, by cultivable and non-cultivable methods. Outdoor data were recorded continuously by the non-cultivable method, using HTSTs samplers (Lanzoni VPPS-2000 samplers Analyzair^®^, Bologna, Italy), in the Gerland area, where no demolition works happened during the study and EHH. They were placed on the building roof respectively 35 m above ground level in Gerland and 10 m in front of the EHH demolition site. *Aspergillaceae* (*Aspergillus* spp. + *Penicillium* spp.) fungal load (AFL), impacted on adhesive tape, placed on a drum, were counted by microscopy every 2 h and expressed as spores per cubic meters every 2 h (spores/m^3^/2-h) or per day (spores/m^3^/day). The number of *Aspergillaceae* spores counted was multiplied by a conversion factor of 0.19, corresponding to a reading of 36.5% of the surface sampled.^3^ HTSTs drums were changed weekly over 42 weeks. Inlet mean flow rate was 10 L.min^-1^ with the non-cultivable method.

With the cultivable method, a daily environmental survey of AFL with agar impact sampler (Air Ideal^®^ 90 mm, bioMérieux) was undertaken outdoor at EHH all around the demolition site (100-L samples). Outdoor samplings were realized four days per week at EHH in the morning (between 10 a.m-12 noon) and afternoon (between 2–4 p.m). Each day, 3 outdoor sites corresponding to the porch of 3 different units were monitored in the morning and afternoon. Each sample was gathered by agar impact sample in 90-mm diameter Petri dishes containing Sabouraud Chloramphenicol agar. Air intake velocity of this agar impact sampler was 100 L/min. Two plates were seeded at each sample site. Each plate was seeded for 1 min, resulting in air volume of 100 L. One of these plates was incubated for 48 h at 37°C to grow thermotolerant *A*. *fumigatus* species [17]. The other plate was incubated for 5 days at 30°C to allow growth of all fungi. Colonies were expressed as spores and colony-forming units per cubic meter (CFU/m^3^) [5].

### Meteorological data

Meteorological parameters were recorded by Meteociel, a meteorological agency, throughout the study period at a station located only a few km from EHH (Fig 1A). Relative humidity (%), temperature (°C), wind direction, rain (mm), atmospheric pressure (hPa) and speed (km.h^-1^) were monitored every hour throughout 2015.

### Statistical data

Data were expressed as medians and interquartile range (IQR). Continuous non-cultivable *Aspergillaceae* data were cumulated per day (over 24 hours). Differences of daily data between EHH and Gerland were compared between the 3 study periods taken pairwise using Wilcoxon test. Non-cultivable AFL data recorded every 2 h were averaged for each day over day time (7 a.m.-7 p.m) and night time (9 p.m.-5 a.m.) and compared between day/night and EHH/Gerland using paired Wilcoxon tests with adjusted p-values for 4 comparisons.

To compare non-cultivable method and cultivable method results for AFL at EHH Wilcoxon test were used. For the non-cultivable method, only data corresponding to the sampling times of cultivable method were used (each day between 10 a.m-12 noon and 2–4 p.m). Data correlations were analyzed with Spearman correlation coefficients: i) between AFL recorded with the two methods, and ii) between AFL *per day* (over 24 h) from both HTSTs (EHH and Gerland).

The impact of meteorological variables on AFL presence estimated in EHH by both methods for the 3 periods was evaluated by fitting logistic regressions with forward selection of variables for each period. In case of significant interaction between 2 meteorological variables, logistic regression was performed in sub-groups, to ease odds ratio (OR) interpretation. Meteorological findings at 11 a.m. and 3 p.m. were paired in the cultivable method with daily samplings in the morning and afternoon. P-values <0.05 were considered to be statistically significant. In case of multiple comparisons, adjusted p-values were computed with the Holm method [18].

## Results

All data were collected over a period of 42 weeks. Table 1 reports medians and IQR of outdoor fungal loads sampled by cultivable and non-cultivable methods during the 3 demolition periods. Differences were observed with each sampler according to sampling site and demolition period.

**Table 1:** *Aspergillaceae* fungal loads in outdoor air sampled by cultivable (CFU/m^3^) and non-cultivable (spores/m^3^/day) methods in the 3 demolition periods.

Site	Sampling method	Periods of demolition work	IQR	Median	Data: n*/**	Total: n
**EHH**	Cultivable	**A**	20	0	216*	511 samples*
		**B**	21	10	118*	
		**C**	80	30	177*	
	Non-cultivable	**A**	13	16	128**	296 days**
		**B**	25	24	62**	
		**C**	10	12	106**	
**Gerland**	Non-cultivable	**A**	35	15	128**	296 days**
		**B**	28	14	62**	
		**C**	10	2	106**	

n*: samples

n**: days

### Description of airborne fungi loads with cultivable and non-cultivable methods

With the cultivable method, a total of 511 air samples were collected outdoor at EHH. The highest median AFL concentrations were observed in Period C (30 CFU/m^3^, IQR:80) during excavation and earthwork. AFL concentration median ranged between 0 (IQR:20) and 30 CFU/m^3^ (IQR:80) in the 3 periods. *Penicillium* median ranged were of 0 CFU/m^3^ in the 3 periods.

Data were collected according to the non-cultivable method for 296 days (42 weeks) at EHH and Gerland. The highest median AFL concentration at EHH was recorded in Period B (median = 23.5 spores/m^3^/day, IQR:24.75) during demolition of the building floors. In contrast, the highest AFL median at Gerland occurred in Period A (median = 15 spores/m^3^/day, IQR:35). AFL medians at EHH and Gerland ranged from 12 (IQR:10) to 23.5 (IQR:24.75) and from 2 (IQR:12) to 15 (IQR:35) spores/m^3^/day, respectively, in the 3 demolition periods.

### Comparisons of *Aspergillaceae* loads in EHH and Gerland

#### Comparison of demolition time periods

**Fig 2:**
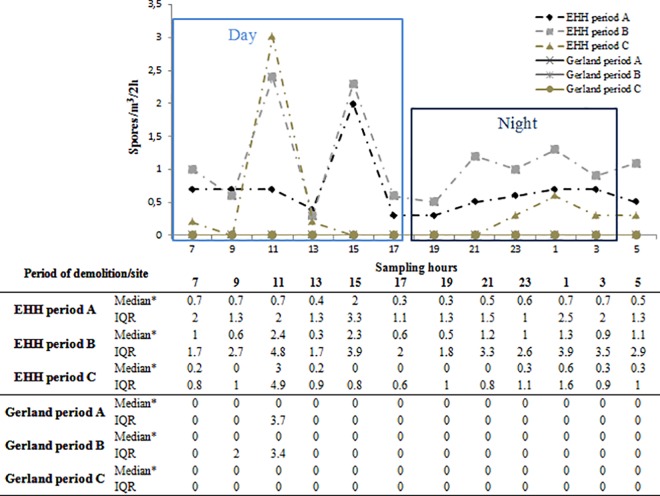
Median of *Aspergillaceae* levels every 2-h by the non-cultivable method at Gerland and EHH during the 3 demolition periods.

Fig 2 reports apparent time period variations in AFL contamination of the non-cultivable spore traps located at EHH and Gerland. AFL medians were observed qualitatively to be much higher at EHH than at Gerland during all study periods. Daily paired AFL differences between the 2 sites (EHH and Gerland) were significantly different between Period A *vs* Period B (median (IQR) of EHH-Gerland differences: 1 (24.3) *vs* 13.5 (31.8), p<10^−4^), Period A *vs* Period C (1 (24.3) *vs* 8 (13.5), p<10^−4^) and Period B *vs* Period C (13.5 (31.8) *vs* 8 (13.5), p = 0.032).

#### Day and night time data per site

At EHH, mean AFL medians were higher during the day (7 a.m.-7 p.m. with every 2-h data) than the night (9 p.m.-5 a.m.) (median (IQR): 1.37 *vs* 1.09 (1.51 *vs* 1.42) spores/m^3^/2-h, p = 0.007). A similar trend was observed at Gerland (median (IQR): 0.57 *vs* 0 (2.14 *vs* 1.48), p = 0.007), attributed to some high AFL (8 peaks>15 spores/m^3^/2-h) in the day time. Statistically significant differences in day time data were evident between EHH and Gerland, with a higher median at EHH (median (IQR): 1.37 (EHH) *vs* 0.57 (1.51 *vs* 2.14) (Gerland) spores/m^3^/2-h, p = 0.010). AFL data were also higher during the night at EHH than at Gerland (median (IQR): 1.09 (EHH) *vs* 0 (1.42 *vs* 1.48) (Gerland) spores/m^3^/2-h, p<10^−7^).

### Cultivable and non-cultivable sampling methods at EHH

**Fig 3:**
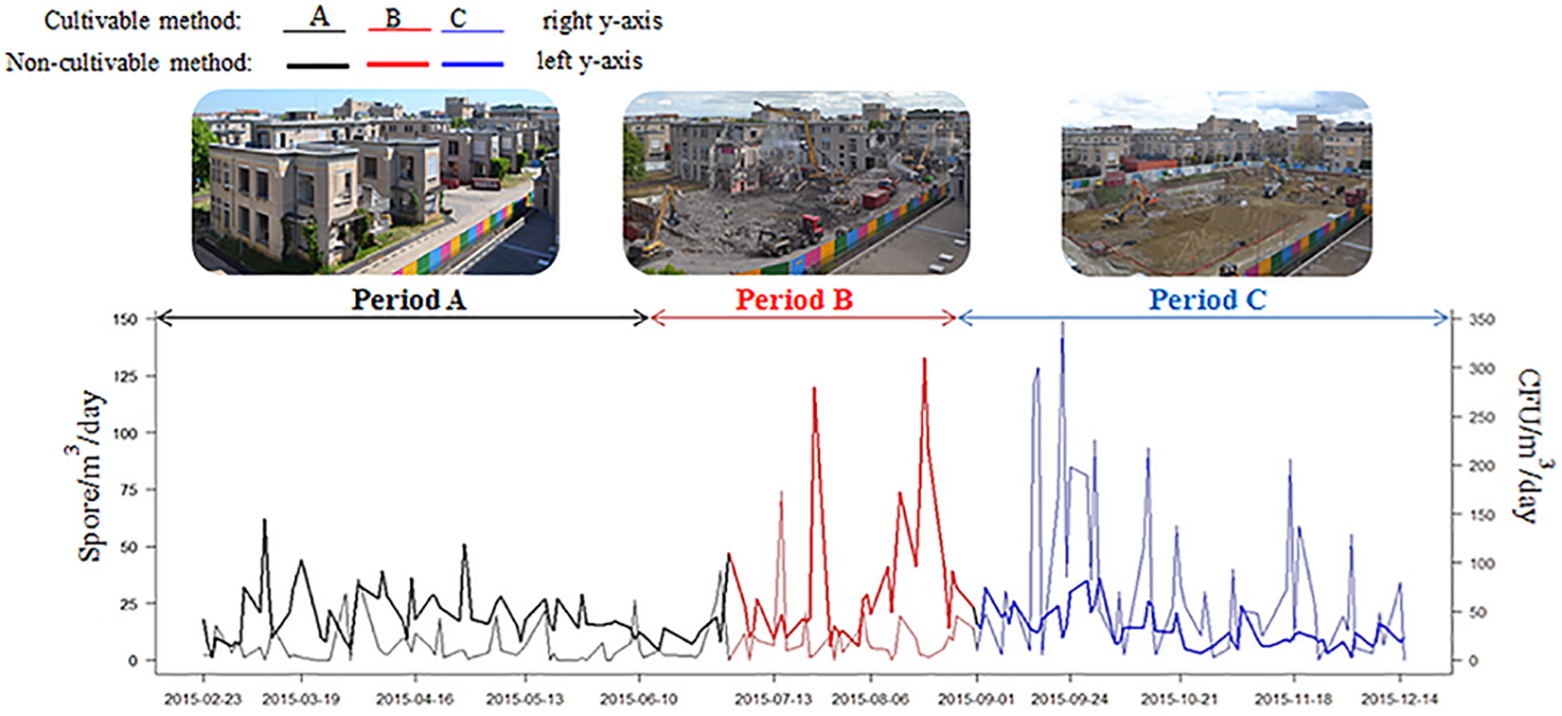
Daily average concentrations of *Aspergillaceae* spores and colonies (spores/m^3^/day and CFU/m^3^) recorded by cultivable and non-cultivable methods during demolition at EHH, in 2015.

Fig 3 reports the mean daily concentrations of AFL colonies and spores (CFU/m^3^ and spores/m^3^/24-h) recorded by both methods during the 3 demolition periods at EHH. A weak correlation between daily AFL recorded by the two methods was observed during Period C (*r* = 0.26, p = 0.0475, n = 109 days), corresponding to excavation and earthwork. During this period, higher peaks were recorded by the cultivable than by the non-cultivable method (Fig 3). No correlation between both methods was observed during Periods A and B (p = 0.6 and p = 0.2, respectively). In Period A, both methods showed similar orders of peak magnitudes. In Period B, higher peaks were observed with the non-cultivable method. The cultivable method produced no significant peaks.

### Evaluation of meteorological impact at EHH

Meteorological variables differed, on average, in the 3 study periods (S1 Table) Table 2 summarizes the results of final, simplified logistic modeling. With the cultivable method, the effects of relative humidity and wind speed on AFL presence were additive in Period A. When relative humidity increased by 10%, the OR of *Aspergillaceae* presence was multiplied by 1.25 (p = 0.035). When wind speed decreased by 10 km/h, the OR of *Aspergillaceae* presence increased by a factor of 2.04 (1/0.49) (p<0.001). In Period B, meteorological variables had no significant effect on AFL. In Period C, when temperature increased by 10°C, the OR of *Aspergillaceae* presence rose 1.93-fold (p = 0.050). With the non-cultivable method, in Period A, in the presence of south or southwestern winds, only temperature had a significant effect on AFL. OR decreased by a factor of 1.41 (1/0.71, p = 0.041) when temperature rose by 10°C. With other wind directions, both temperature and atmospheric pressure had significant, additive effects on *Aspergillaceae* presence whose OR increased 1.79-fold when atmospheric pressure rose by 10 hPa (p<10^−4^). The effect of temperature was similar (OR = 0.71, p<10^−3^). In Periods B and C, only relative humidity had a significant impact on *Aspergillaceae* whose OR increased by a factor of 1.21 (p<10^−4^) and decreased by a factor of 1.08 (1/0.92, p = 0.03), respectively, when relative humidity rose by 10%.

**Table 2:** Impact of meteorological variables on AFL contamination measured at EHH by cultivable and non-cultivable methods and estimated by final univariate or multivariate logistic regression after forward meteorological variable selection.

Sampling method	Demolition periods	n	Explicative meteorological variables	Adjusted^1^ OR	95% CI	p-value
**Cultivable method**	**A**	216	Relative humidity	1.25	1.02–1.54	0.035
			Wind speed	0.49	0.34–0.72	<10^−3^
	**B**	118				
	**C**	177	Temperature	1.93	1.001–3.8	0.050
**Non-cultivable method**	**A**	382	Temperature*	0.71	0.50–0.99	0.041
	*Analysis of*	1,154	Temperature	0.71	0.58–0.86	<10^−3^
	*2 subgroups*		Atmospheric pressure*	1.71	1.34–2.19	<10^−4^
	**B**	743	Relative humidity	1.21	1.10–1.34	<10^−4^
	**C**	1,271	Relative humidity	0.92	0.86–0.99	0.030

^1^Adjusted OR in case of at least 2 explicative variables

*Only univariate regression in the subgroup with south or southwestern winds

OR is given for 10-unit augmentation in case of continuous meteorological variables

## Discussion

This study revealed: i) the capacity of HTSTs to detect *Aspergillaceae* aero-contamination of the surrounding environment, ii) weak correlation (trend) between AFL recorded by cultivable and non-cultivable methods, and iii) consistent influence of environmental factors, such as demolition work and meteorological variables, on AFL.

HTSTs seem to be able to detect variations of outdoor AFL, as described in previous studies [4,9,11,15]. Higher AFL was detected at EHH which underwent major demolition compared to Gerland, the control site. These data highlighted the importance to be careful in protecting immunocompromised patients when they are outside units during demolition at hospital. Similarly, higher AFLs were recorded at EHH during days than during nights. Even if AFL at EHH were lower during the night than the day, aerocontamination still stayed higher than at Gerland area. So period of opening windows during nights have to be realised with caution, during limited times.

These samplers were highly influenced by environmental conditions and factors, as proved by differences observed between the Gerland area (control) and EHH [16,19]. The present study identified demolition work as the potential source of AFL. At EHH site, some peaks were observed in the morning and/or the afternoon. A possible explanation to those peaks could be the presence of high activity of the demolition site at these times. Distinct AFL peaks according to each demolition period were seen with both sampling methods.

To the best of the authors’ knowledge, this is the first report of weak but significant correlation between both sampling methods of AFL monitoring during earthwork (Period C). As AFL recorded by cultivable sampling was found to be higher in Period C, the results confirmed previous studies which suggested that high concentrations of spores and numerous sampling measures are needed to find statistical relationships between both methods [15]. The kind of demolition works could explain these high AFL levels. Excavation and earthwork were done at ground level or just below, at the height where the cultivable sampler was recording. In contrast, weak AFL contamination was monitored by the non-cultivable method during this period because of the height separating the collector and the demolition works. The absence of correlation in other periods could be explained by the location/height of the samplers according to type of demolition work undertaken. Some experts suggest that particles concentrations decrease with height, possibly owing to dispersion phenomena [4,19]. During Period A, only inside demolition work was done. The absence of correlation may be due to low spore concentrations observed with the cultivable method, possibly because of its position between buildings [20] In Period B, the non-cultivable method showed higher AFL than the cultivable method, explained by type of demolition work occurring during this period, i.e., demolition of building floors. Demolition work was done approximately 15 m above ground level, corresponding to the height at which the HTSTs were located. The cultivable sampler located at ground level was unable to detect variations due to these demolition works. It may explain the lack of correlation between both methods. In the present study, the type of demolition work highly impacted AFL monitored by both methods because of differences in height.

Meteorological effects on AFL data were found consistent with literature [11, 15]. *Aspergillus* species are known to gain optimal growth between 30 and 35°C with average humidity of 70%. Temperature and relative humidity were closely related to AFL growth and reproduction capacity. Each demolition period highly corresponded to a season, heavily influencing data analysis and could help future studies in predicting and modeling AFL peaks.

The main strength of this study resides in the numerous data obtained with both methods, allowing to answer important questions in environmental monitoring during construction works and helping infection control staff in planning precautions for immunocompromised patients. It does, however, have some limitations: the demolition periods were concomitant with the seasons which could have induced seasonality bias in analysis. No baseline period without demolition work at EHH could be studied.

It is known that air flow from outdoors can be found indoors, increasing the risk for the patient to develop IA [8]. To limit exposure, rapid detection of contamination peaks is needed to implement adequate prevention measures and policies. This study permit to confirm the careful attention needed to be taken in protecting immunocompromised patients when they are outside units during demolition. Analysis of day and night AFL variations showed that although lower AFL were found during the night at EHH, loads still staid higher than control area. So, to ensure safety of patients, limited period of opening windows during night have to be recommended. Furthermore, this study give some new insights to improve environmental monitoring guidelines by showing importance of samplers height position in regards to king of demolition work applied.

Further studies, including both methods at different ground levels, are needed to better evaluate sensitivity and reliability as warning systems. Finally, our study determined that outdoor HTSTs are promising tool for outdoor AFL monitoring during major demolition works in hospital, helping clinical infection control practitioners to rapidly implement control measures.

## Supporting information

S1 Table Relationship between each meteorological variable and the 3 study periods. ***p<0.001; **p< 0.01. Multiple comparisons were made between study periods for each meteorological variable.
(DOCX)
