# Supplementary material for: Evaluation of Hirst-type spore traps in outdoor Aspergillaceae monitoring during large demolition work in hospital
Source: PLoS One. 2018 Jan 18;13(1):e0191135. doi: 10.1371/journal.pone.0191135 (PMC5773167; doi:10.1371/journal.pone.0191135)
Supplement: S1 Table — ***p<0.001; **p< 0.01. Multiple comparisons were made between study periods for each meteorological variable. (DOCX) [file pone.0191135.s001.docx]

**Supporting informations:**

**Table S1**: Relationship between each meteorological variable and the 3 study periods

| **Meteorological variables** | **Variables** | **Period A** | **Period B** | **Period C** | **Global  p-value** | **Adjusted p-values** | | |
| --- | --- | --- | --- | --- | --- | --- | --- | --- |
|  |  |  |  |  |  | **A-B** | **A-C** | **B-C** |
| **Frequency** | n | 3,069 | 1,488 | 2,543 | *** |  |  |  |
|  |  |  |  |  |  |  |  |  |
| **Temperature (°C)** | Mean (SD) | 14.8 (6.9) | 24.6 (5.8) | 11.6 (5.7) | *** | *** | *** | *** |
| **Humidity (%)** |  | 61.8 (18.4) | 48.6 (19.5) | 76.5 (15.6) | *** | *** | *** | *** |
| **Atmospheric pressure (hPa)** |  | 1,019.2 (6.0) | 1,015.6 (3.4) | 1,021.5 (7.6) | *** | *** | *** | *** |
|  |  |  |  |  |  |  |  |  |
| **Wind speed (km/h)** | Median (range) | 9 (0-117) | 11 (0-48) | 6 (0-70) | *** | *** | *** | *** |
|  |  |  |  |  |  |  |  |  |
| **Rain (Yes/No)** | n (%) | 236 (7.7) | 47 (3.2) | 140 (5.5) | *** | *** | ** | ** |
| **South or southwestern wind (Yes/No)** | n (%) | 751 (24.5) | 502 (33.7) | 587 (23.1) | *** | *** | 0.23 | *** |

***p<0.001; **p< 0.01. Multiple comparisons were made between study periods for each meteorological variable
